# Supplementary material for: Electronic health record-based genome-wide meta-analysis provides insights on the genetic architecture of non-alcoholic fatty liver disease
Source: Cell Rep Med. 2021 Nov 3;2(11):100437. doi: 10.1016/j.xcrm.2021.100437 (PMC8606899; doi:10.1016/j.xcrm.2021.100437)
Supplement: Document S1. Figures S1–S3 [file mmc1.pdf]

Cell Reports Medicine, Volume *2*

## Supplemental information

# Electronic health record-based genome-wide meta-analysis provides insights on the genetic architecture of non-alcoholic fatty liver disease

**Nooshin Ghodsian, Erik Abner, Connor A. Emdin, Émilie Gobeil, Nele Taba, Mary E. Haas, Nicolas Perrot, Hasanga D. Manikpurage, Éloi Gagnon, Jérôme Bourgault, Alexis St-Amand, Christian Couture, Patricia L. Mitchell, Yohan Bossé, Patrick Mathieu, Marie-Claude Vohl, André Tchernof, Sébastien Thériault, Amit V. Khera, Tõnu Esko, and Benoit J. Arsenault**

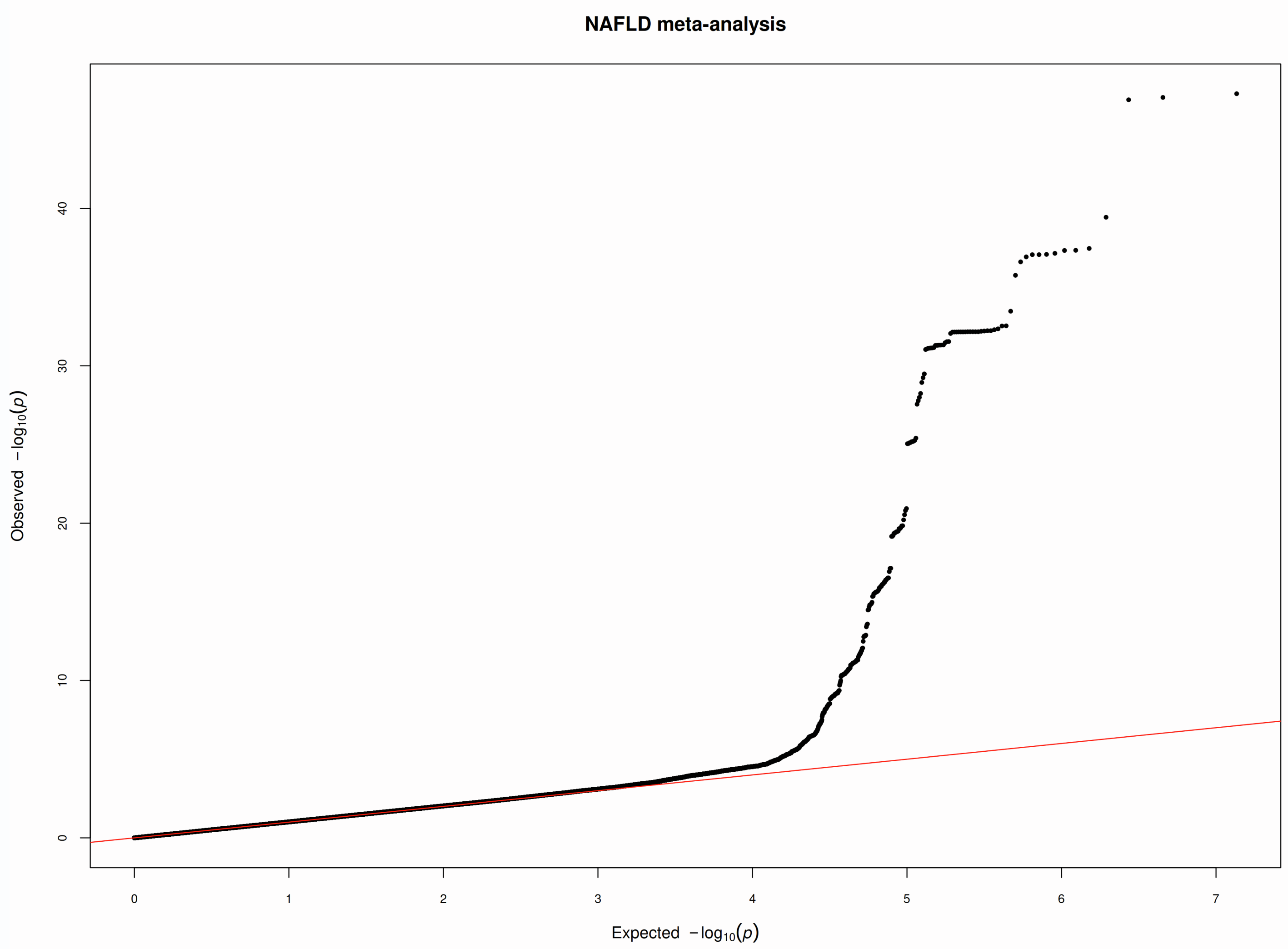


**Supplementary Figure 1:** Quantile-quantile plot of the NAFLD GWAS meta-analysis.

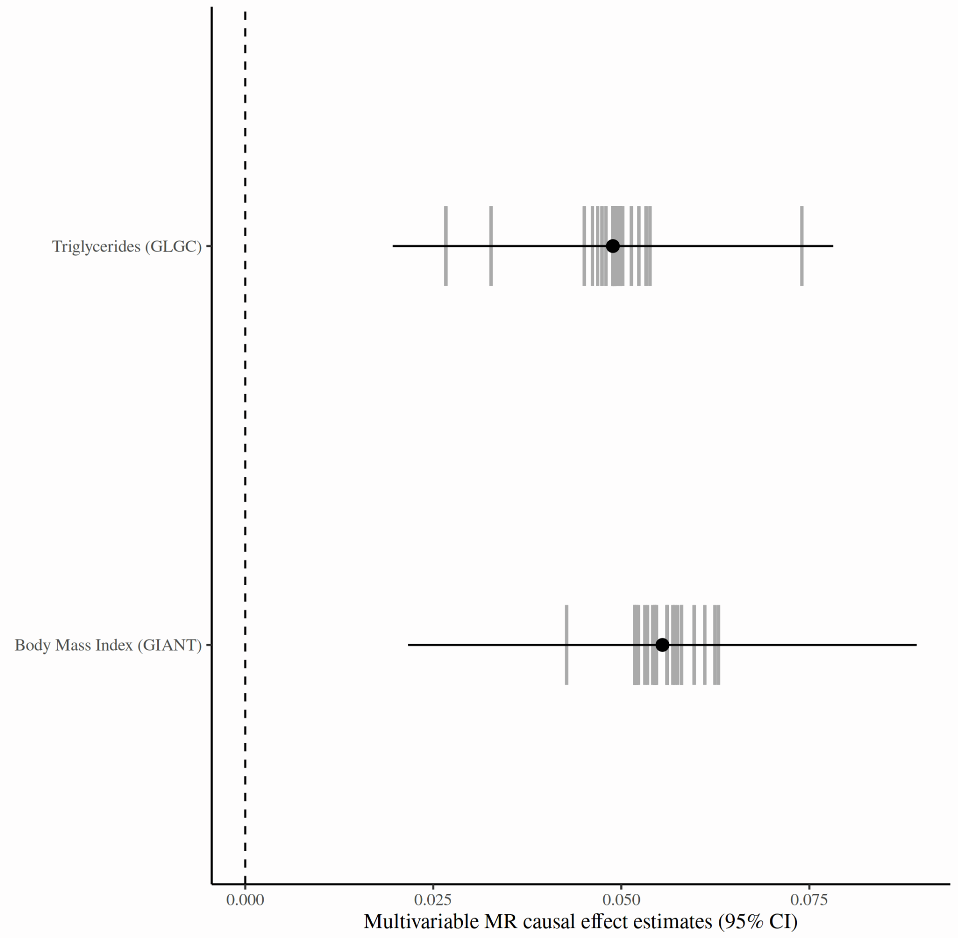


**Supplementary Figure 2:** Coefficient plot for the risk factors used to create the prior. For each risk factor, the multivariable causal estimate and the 95% interval from multivariable Mendelian randomization model using all chromosome (black dots and bars) as well as the 22 per-chromosome estimates (grey bars) are presented.

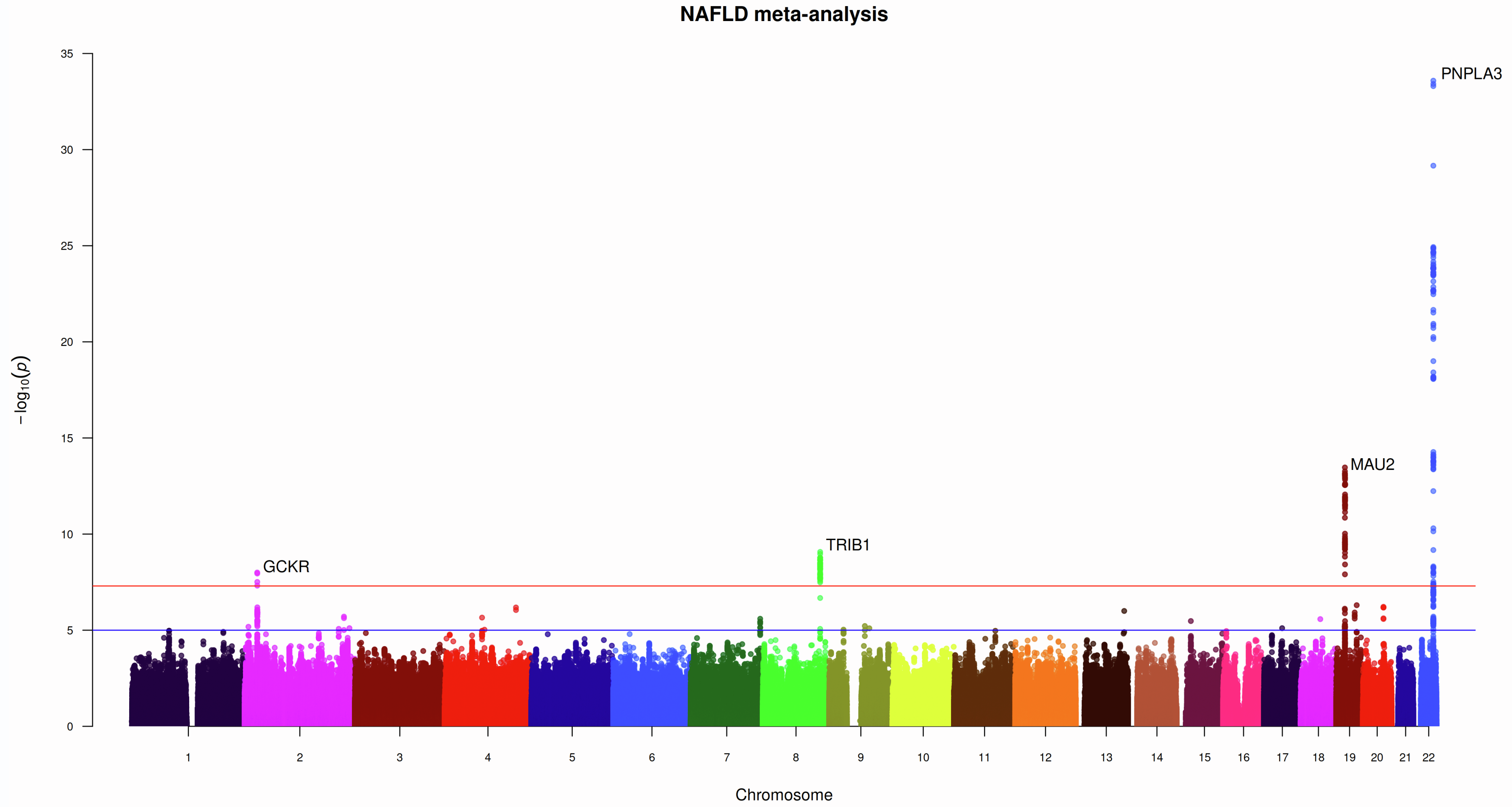


**Supplementary Figure 3:** Results of body-mass index-adjusted meta-analysis of genome-wide association studies.
