# Supplementary material for: Electronic health record-based genome-wide meta-analysis provides insights on the genetic architecture of non-alcoholic fatty liver disease
Source: Cell Rep Med. 2021 Nov 3;2(11):100437. doi: 10.1016/j.xcrm.2021.100437 (PMC8606899; doi:10.1016/j.xcrm.2021.100437)
Supplement: Document S2. Article plus supplemental information [file mmc7.pdf]

Cell Reports
Medicine

Article

Electronic health record-based genome-wide meta-analysis provides insights on the genetic architecture of non-alcoholic fatty liver disease

Graphical abstract

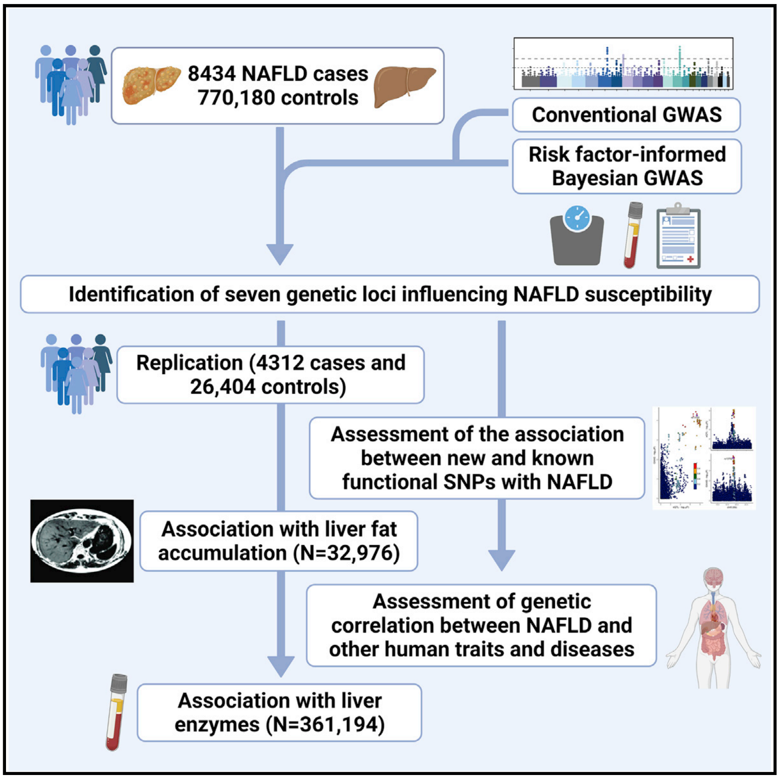

Highlights

- This analysis identifies 5 genetic loci for non-alcoholic fatty liver disease
- Non-alcoholic fatty liver disease loci are *GCKR*, *TR1B1*, *TM6SF2*, *APOE,* and *PNPLA3*
- Adipose tissue *LPL* expression may influence non-alcoholic fatty liver disease
- The *FTO* genotype may affect non-alcoholic fatty liver disease susceptibility

Authors

Nooshin Ghodsian, Erik Abner, Connor A. Emdin, ..., Amit V. Khera, Tõnu Esko, Benoit J. Arsenault

Correspondence

benoit.arsenault@criucpq.ulaval.ca

In brief

To gain insight into the genetic architecture of non-alcoholic fatty liver disease (NAFLD), Ghodsian et al. performed a genome-wide meta-analysis of electronic health record-documented NAFLD and identify 7 potential susceptibility loci for this disease (located at or near *GCKR*, *TR1B1*, *LPL*, *FTO*, *MAU2*/*TM6SF2*, *APOE*, and *PNPLA3*).

Ghodsian et al., 2021, Cell Reports Medicine 2, 100437
November 16, 2021 © 2021 The Author(s).
https://doi.org/10.1016/j.xcrm.2021.100437

Cell Reports Medicine

CellPress
OPEN ACCESS

Article

Electronic health record-based genome-wide meta-analysis provides insights on the genetic architecture of non-alcoholic fatty liver disease

Nooshin Ghodsian,[1] Erik Abner,[2] Connor A. Emdin,[3,4] Émilie Gobeil,[1] Nele Taba,[2,5] Mary E. Haas,[3,6] Nicolas Perrot,[1] Hasanga D. Manikpurage,[1] Éloi Gagnon,[1] Jérôme Bourgault,[1] Alexis St-Amand,[1] Christian Couture,[1] Patricia L. Mitchell,[1] Yohan Bossé,[1,7] Patrick Mathieu,[1,8] Marie-Claude Vohl,[9,10] André Tchernof,[1,10] Sébastien Thériault,[1,11] Amit V. Khera,[3,4,12] Tõnu Esko,[2] and Benoit J. Arsenault[1,13,14,*]

[1]Centre de Recherche de l'Institut Universitaire de Cardiologie et de Pneumologie de Québec, Québec, QC, Canada
[2]Estonian Genome Center, Institute of Genomics, University of Tartu, Tartu, Riia 23b, 51010, Estonia
[3]Program in Medical and Population Genetics, Broad Institute, Cambridge, MA, USA
[4]Department of Medicine, Harvard Medical School, Boston, MA 02114, USA
[5]Institute of Molecular and Cell Biology, University of Tartu, Tartu, Riia 23, 51010, Estonia
[6]Department of Molecular Biology, Department of Medicine, Massachusetts General Hospital, Boston, MA 02114, USA
[7]Department of Molecular Medicine, Faculty of Medicine, Université Laval, Québec, QC, Canada
[8]Department of Surgery, Faculty of Medicine, Université Laval, Québec, QC, Canada
[9]Centre NUTRISS, Institut sur la Nutrition et les Aliments Fonctionnels, Université Laval, Québec, QC, Canada
[10]School of Nutrition, Université Laval, Québec, QC, Canada
[11]Department of Molecular Biology, Medical Biochemistry and Pathology, Faculty of Medicine, Université Laval, Québec, QC, Canada
[12]Center for Genomic Medicine, Department of Medicine, Massachusetts General Hospital, Boston, MA 02114, USA
[13]Department of Medicine, Faculty of Medicine, Université Laval, Québec, QC, Canada
[14]Lead contact
*Correspondence: benoit.arsenault@criucpq.ulaval.ca
https://doi.org/10.1016/j.xcrm.2021.100437

SUMMARY

Non-alcoholic fatty liver disease (NAFLD) is a complex disease linked to several chronic diseases. We aimed at identifying genetic variants associated with NAFLD and evaluating their functional consequences. We performed a genome-wide meta-analysis of 4 cohorts of electronic health record-documented NAFLD in participants of European ancestry (8,434 cases and 770,180 controls). We identify 5 potential susceptibility loci for NAFLD (located at or near *GCKR*, *TR1B1*, *MAU2/TM6SF2*, *APOE*, and *PNPLA3*). We also report a potentially causal effect of lower *LPL* expression in adipose tissue on NAFLD susceptibility and an effect of the *FTO* genotype on NAFLD. Positive genetic correlations between NAFLD and cardiometabolic diseases and risk factors such as body fat accumulation/distribution, lipoprotein-lipid levels, insulin resistance, and coronary artery disease and negative genetic correlations with parental lifespan, socio-economic status, and acetoacetate levels are observed. This large GWAS meta-analysis reveals insights into the genetic architecture of NAFLD.

INTRODUCTION

Non-alcoholic fatty liver disease (NAFLD) is one of the most prevalent chronic liver diseases.[1,2] According to recent estimates, ~25% of the adult population worldwide may have NAFLD.[3,4] This disease has been predicted to become the most frequent indication for liver transplantation in Western countries by 2030.[5] NAFLD is a progressive liver disease with potential consequences for several other chronic disorders such as cardiovascular disease (CVD) (the leading cause of death in patients with NAFLD),[6–9] type 2 diabetes (T2D),[10,11] dyslipidemia,[12] and other extrahepatic manifestations such as chronic kidney disease[13] and gastrointestinal neoplasms.[14]

To better understand the etiology of complex diseases such as NAFLD and to develop therapies that may help patients with this disease living longer and healthier, the genetic architecture of NAFLD needs to be better understood. Although genome-wide association studies (GWASs) have identified genetic variants associated with liver fat accumulation,[15,16] liver enzymes,[17] and different forms of liver diseases,[18,19] less than a handful of small GWASs sought to identify genetic variants associated with a clinical diagnosis of NAFLD. The GWAS of the Electronic Medical Records and Genomics (eMERGE) network included 1,106 NAFLD cases and 8,571 controls identified only 1 NAFLD susceptibility locus (*PNPLA3*). The NAFLD GWAS of the UK Biobank included 1,664 NAFLD cases and 400,055 controls identified only 2 regions robustly associated with NAFLD (*PNPLA3* and *PBX4/TM6SF2*). The UK Biobank analysis did not exclude participants with secondary causes of NAFLD (e.g., hepatitis, alcoholism) and used a rather vague definition

Check for updates

© 2021 The Author(s).
This is an open access article under the CC BY license (http://creativecommons.org/licenses/by/4.0/).

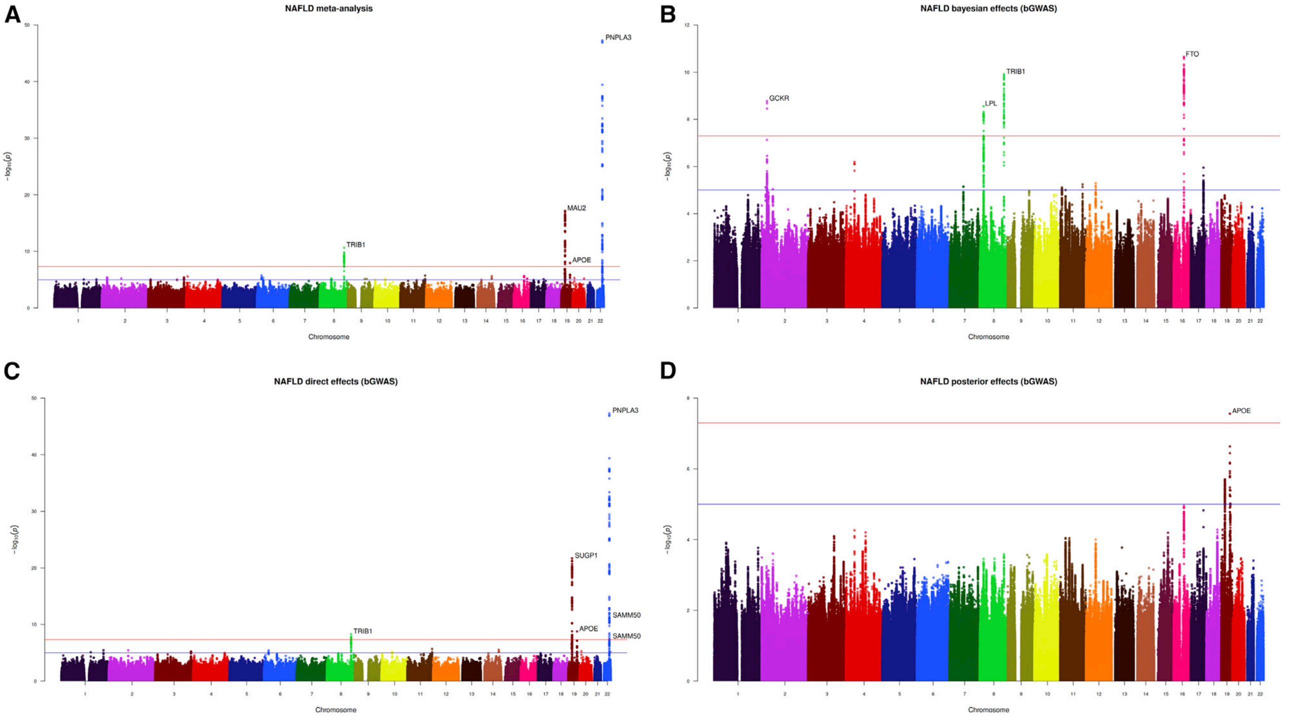

Figure 1. Main results of the meta-analysis of genome-wide association studies (GWASs)

(A) Manhattan plot depicting single-nucleotide polymorphisms (SNPs) associated with non-alcoholic fatty liver disease in the GWAS meta-analysis of the eMERGE, FinnGen, UK Biobank, and Estonian Biobank cohorts. Identification of genetic variants linked with NAFLD via a risk factor-informed Bayesian GWAS based on (B) Bayes Factors (BFs), (C) direct effects, and (D) posterior effects. Genetic loci harboring SNPs associated with NAFLD ($p < 5.0e-8$) are shown.

of NAFLD (phecode 571.5: other forms of nonalcoholic liver disease). Genetic variation at these 2 loci is also associated with NAFLD in the data freeze #4 of the FinnGen cohorts (651 NAFLD cases and 176,248 controls).

Here, we present the results of a meta-analysis of electronic health record (EHR)-based GWASs to identify genetic variants associated with NAFLD. This analysis included GWAS summary statistics from the eMERGE and FinnGen cohorts, an updated NAFLD GWAS in the UK Biobank (2,558 cases and 395,241 controls), and a new GWAS performed in the Estonian Biobank (4,119 cases and 190,120 controls), for a total of 8,434 NAFLD cases and 770,180 controls.

RESULTS

Identification of genetic variants associated with NAFLD

To identify genetic variants associated with NAFLD, we performed 2 new GWASes in the UK Biobank and Estonian Biobank and performed a meta-analysis of 4 cohorts (UK Biobank, Estonian Biobank, eMERGE, and FinnGen), totaling 8,434 NAFLD cases, all identified through EHRs, and 770,180 controls. We identified 4 genetic loci that harbored at least 1 SNP that passed the genome-wide significance threshold of p £ 5 × 10^{-8} (*TRIB1*, *MAU2* [*TM6SF2*], *APOE*, and *PNPLA3*). Figure 1A presents the Manhattan plot of the NAFLD GWAS meta-analysis identifying genetic regions with a p value for association with NAFLD £5 × 10^{-8}. The associated quantile-quantile plot is presented in Figure S1. The genomic inflation factor (λ) was 1.02 and the linkage disequilibrium score regression (LDSC) intercept was 1.002. To identify potentially new relevant NAFLD genetic loci, we used a Bayesian approach (bGWAS) recently described by Mounier and Kutalik.[20] This method seeks to identify new variants associated with complex diseases using inference from risk factors of these diseases. By leveraging GWAS summary statistics from risk factors likely causally associated with NAFLD in a previous magnetic resonance imaging (MRI) study[21] (body mass index [BMI] and triglyceride levels) as priors, this analysis revealed genetic variation at 3 additional loci (*GCKR*, *LPL*, and *FTO*) associated with NAFLD (Table S1; STAR Methods). Figure S2 presents the multivariable causal effect estimates for the 2 risk factors (BMI and triglycerides) used to create the prior. Variation at these loci act through selected NAFLD risk factors on Bayes factors, meaning that these SNPs are acting on NAFLD through their effect on risk factors (Figure 1B), rather than through direct effects (Figure 1C) or posterior effects (Figure 1D) (i.e., not acting through selected risk factors). The association of lead SNPs at these loci with NAFLD as well as those from the conventional GWAS are presented in Table S2 in each cohort separately and in the GWAS meta-analysis. Because some of these SNPs showed evidence of heterogeneity, p values are presented from fixed effects and random effects meta-analysis. Through a combination of conventional GWAS and risk factor-informed GWAS, our analysis identified genetic variation at 7 loci that may influence susceptibility to NAFLD.

Table 1. Association of previously identified functional variants linked with liver diseases in the present genome-wide association study

						Association with NAFLD		
Gene	CHR	SNP	Impact on protein	Minor allele	Major allele	β (minor allele)	SE	p
MTARC1	1	rs2642438	missense (p.A165T)	A	G	−0.0674	0.0178	1.54E−4
GCKR	2	rs1260326	missense (p.P446L)	T	C	0.0755	0.0167	5.98E−6
HSD17B13[a]	4	rs72613567	splice variant	C	G	−0.0304	0.0186	1.02E−1
MBOAT7	19	rs641738	linked to 3' UTR	T	C	0.0519	0.0164	1.53E−3
APOE	19	rs429358	missense (p.R130C)	C	T	−0.1366	0.0239	1.14E−8
TM6SF2	19	rs58542926	missense (p.E167K)	T	C	0.2676	0.0320	6.90E−17
PNPLA3	22	rs738409	missense (p.I148M)	G	C	0.2869	0.0198	1.23E−47

[a]The effect of a SNP in linkage disequilibrium (r^2 = 0.96) with this variant (rs10433879) is presented.

Impact of the 7 variants on NAFLD after accounting for obesity

To determine whether these 7 SNPs were associated with NAFLD independently of obesity, we performed another GWAS meta-analysis using the same models described in the Method details section but adding BMI as a covariate. The GWAS from eMERGE already provided summary statistics adjusted for BMI. Because BMI was not available for every participant of the UK and especially the Estonian Biobank, we performed another GWAS in slightly fewer individuals in the UK Biobank (2,541 cases and 394,053 controls) and in the Estonian Biobank participants with available BMI values (2,817 cases and 133,909 controls). The total number of NAFLD cases for this analysis was 6,464 and the total number of controls was 536,533. The Manhattan plot of this GWAS meta-analysis is presented in Figure S3. The impact of the 7 SNPs on NAFLD in BMI-adjusted analyses are presented in Table S3. The effect of the 7 variants on NAFLD appeared to remain in the same range, with the exception of *FTO*, which was no longer statistically significant after adjusting for BMI. Interestingly, the association between the variant at the *GCKR* locus (rs1260326) became associated with NAFLD, with a p value below the GWAS significance threshold of £5 × 10^{-8}. This analysis did not reveal any new NAFLD susceptibility loci beyond the variant at the *GCKR* locus.

Evaluation of the functionality of variants associated with NAFLD

Some of the top variants linked with NAFLD in this analysis may have functional consequences. For instance, the rs1260326 at *GCKR* is a missense variant (p.P446L). The rs1260326 at *APOE* is also a missense variant (p.R130C). The lead variant at *MAU2/TM6SF2* rs73001065 is in linkage disequilibrium (r^2 = 0.90) with the missense variant p.E167K at *TM6SF2*, and the lead variant at *PNPLA3* is in high linkage disequilibrium (r^2 = 0.98) with the missense variant p.I148M at *PNPLA3*. Table 1 presents the details of these results as well as the effect of other previously associated variants with NAFLD (p.A165T at *MTARC1*, a splice variant *HSD17B13*, and another variant at *MBOAT7*). This analysis confirmed previous NAFLD functional variants at *MTARC1* and *MBOAT7*, but not at *HSD17B13*. Genetic variation at the *PNPLA3*, *TM6SF2*, and *GCKR* have been linked with NAFLD-related traits in previous studies.[15,22,23] Recent studies identified *APOE*, *TR1B1*, and *FTO* as potential new loci for liver enzymes.[24,25] Our study extends the results of these studies by linking variation at these loci with a clinical diagnosis of NAFLD and identifies *LPL* as a potential new susceptibility locus for NAFLD. Interestingly, the minor allele (C) at rs13702 associated here with protection against NAFLD has been predicted to disrupt a microRNA recognition element seed site for human microRNA miR-410, resulting in higher *LPL* expression.[26] We therefore sought to determine whether genetically predicted *LPL* expression was associated with NAFLD. We performed a transcriptome-wide association study for NAFLD to map genetically regulated genes from the Genotype Tissue Expression (GTEx, version 8) consortium[27] with NAFLD using S-PrediXcan. This analysis did not reveal new NAFLD genes outside those that had a genome-wide signal such as *PNPLA3* and *TM6SF2* (data not shown). Genetically predicted *LPL* expression could be estimated in 11 tissues. The association between genetically predicted *LPL* expression in these 11 tissues and NAFLD is presented in Table S4. This analysis suggests a negative association between genetically predicted *LPL* expression in subcutaneous adipose tissue and NAFLD (p = 3.1e−4). The LocusCompare plot (Figure 2) further suggests shared genetic etiology at this locus with the rs13702 variant being significantly associated with both subcutaneous adipose tissue expression of *LPL* and NAFLD.[28] In summary, most of the 7 SNPs identified in this analysis or SNPs in close proximity may be considered functional SNPs.

Association of variants associated with NAFLD with NAFLD-related phenotypes

We investigated the effect of these variants in another cohort and with NAFLD-related traits such as liver fat accumulation and liver enzymes in the UK Biobank. In the Mass General Brigham Biobank, 4,312 patients with non-alcoholic steatohepatitis (NASH) or NAFLD (diagnosed by computed tomography and/or MRI) were compared to 26,404 controls. The direction of the effects of the 7 SNPs were concordant with those observed in the GWAS meta-analysis. All SNPs were significantly associated with NAFLD in the Mass General Brigham Biobank, with the exception of the variants at the *FTO* and at the *LPL* loci (Table S5). Liver fat accumulation in the UK Biobank was quantified via machine learning of abdominal MRI images, as previously described.[29] We analyzed liver fat accumulation as a continuous

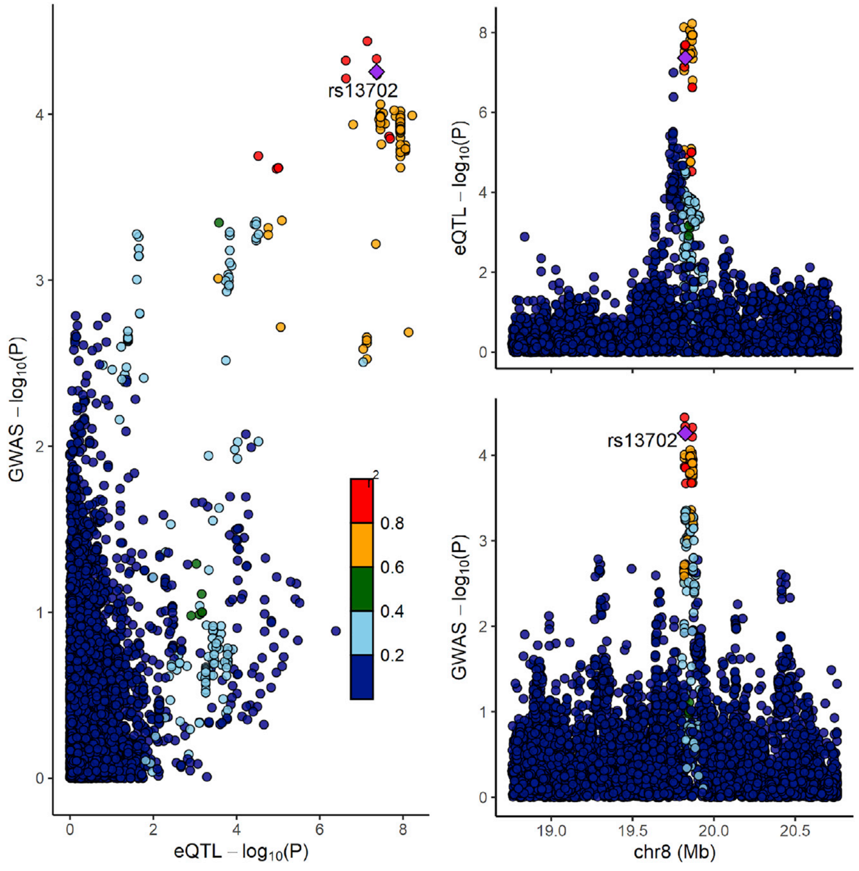

Figure 2. Shared genetic etiology at the *LPL* locus

LocusCompare plot depicting colocalization of the top SNPs associated with subcutaneous adipose tissue *LPL* expression and NAFLD. Each dot represents a SNP at the *LPL* locus. In the left panel, these SNPs are plotted to represent their effect on *LPL* expression (top right) against their effect on NAFLD (bottom right).

trait in 32,976 study participants. The direction of the effects of the 7 SNPs on liver fat accumulation was concordant with those observed in the GWAS meta-analysis, and all SNPs were significantly associated with liver fat accumulation, with the exception of the variant at the *LPL* locus (Table S5). Finally, the association between the 7 variants associated NAFLD with the liver enzymes ALT (alanine aminotransferase), AST (aspartate aminotransferase), GGT (γ-glutamyl transferase), and ALP (alkaline phosphatase) was investigated in 361,194 participants of the UK Biobank. Results presented in Table S5 suggest that all of the variants were positively associated with liver enzymes, except that the variant at *GCKR* was not associated with ALT levels, the variant at *APOE* was not associated with AST levels, and the variant at *PNPLA3* was not associated with GGT levels. Variants at the *GCKR*, *LPL*, *TRIB1*, and *APOE* were positively associated with ALP levels, the variant at *FTO* was not associated with ALP levels, and the variants at *MAU2/TM6SF2* and *PNPLA3* were negatively associated with ALP levels. Overall, the results of this analysis suggest that the 7 variants associated with NAFLD are associated with NAFLD-related traits such as liver fat accumulation and/or liver enzymes.

Association of NAFLD with human metabolic and phenotypic traits

We performed cross-trait genetic correlation analyses between NAFLD and 240 human traits centralized in the LD Hub database. LD Hub includes GWAS publicly available summary statistics on hundreds of human traits and enables the assessment of LD score regression among those traits. The results presented in Figure 3 show high levels of genetic correlation between NAFLD and cardiometabolic traits and diseases such as obesity, insulin resistance, triglycerides, coronary artery disease (CAD), T2D, and negative genetic correlation with parental lifespan, education, and the ketone body acetoacetate.

DISCUSSION

We performed 2 genome-wide association studies for NAFLD in the UK Biobank and in the Estonian Biobank and combined these results with those of 2 publicly available NAFLD GWASs (from the eMERGE network and FinnGen). This GWAS meta-analysis included 8,434 NAFLD cases available via EHRs and 770,180 controls, making it the largest genome-wide analysis for a clinical diagnosis of NAFLD. In combination with a risk factor-informed bGWAS, this analysis identified 2 known susceptibility loci for NAFLD (*TM6SF2* and *PNPLA3*) and 5 potentially new candidate genetic regions for a clinical diagnosis NAFLD based on EHRs (*GCKR*, *TRIB1*, *LPL*, *FTO*, *APOE*).

Our conventional GWAS analyses (adjusted for BMI or not) report that variation at the *GCKR*, *TRIB1*, *MAU2/TM6SF2*, *APOE*, and *PNPLA3* loci may be linked to NAFLD. While genetic variants at these loci have been associated with some liver phenotypes,[16,18,22,23] this GWAS meta-analysis revealed important information on the genetic architecture of NAFLD. Using bGWAS, our study identified known and potentially new loci for NAFLD (*LPL* and *FTO)* that may be associated with NAFLD through their effects on NAFLD risk factors (BMI and triglycerides). A recent preprint identified a variant at the *FTO* locus as a susceptibility locus for having high ALT levels in the Million Veteran Program[30] Although the biological relevance of variation at the *FTO* locus is still a matter of debate, *FTO* is a well-characterized genetic locus for obesity.[31] Upon adjusting for BMI, the association between the variant at the *FTO* locus was no longer significantly associated with NAFLD, confirming that the effect of this variant on NAFLD is dependent on its effect on body weight. Although variants at the *GCKR* locus were not associated with NAFLD in the main analysis, the bGWAS analysis

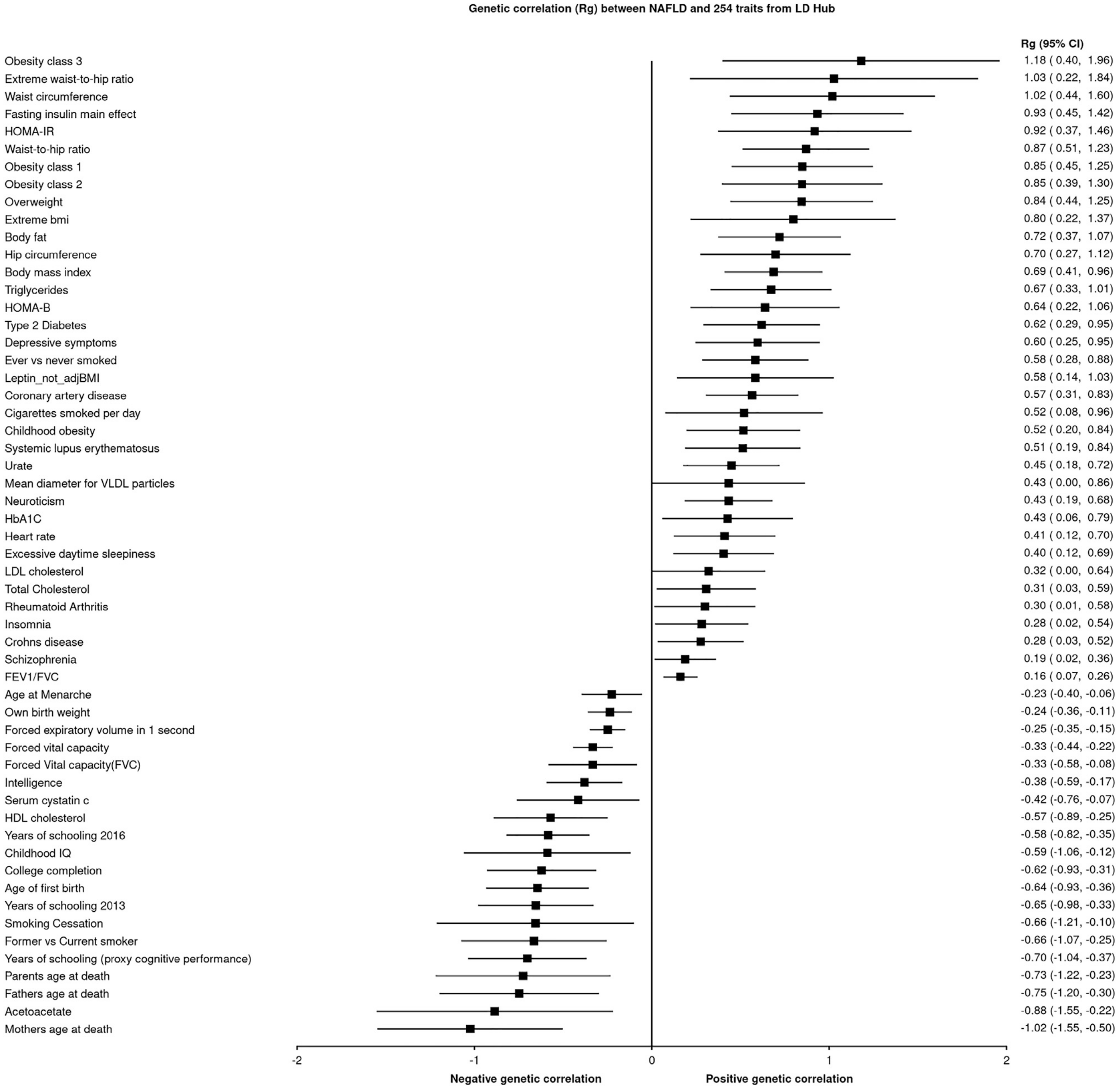

Figure 3. Results of the LD regression analysis between NAFLD and other human diseases and traits

LD regression analyses were performed in LD Hub to test the genetic correlation of NAFLD with 240 human diseases and traits. Statistically significant ($p < 0.05$) genetic correlation coefficients (Rg) and their 95% confidence intervals are presented. adjBMI, adjusted for body mass index; FEV1/FVC, forced expiratory volume in 1 s/forced vital capacity; HOMA-IR, homeostatic model of insulin resistance; VLDL, very-low-density lipoproteins.

and the conventional GWAS adjusted for BMI identified GCKR as a susceptibility locus for NAFLD. Other studies reported an association of variants at the *GCKR* loci and liver fat accumulation[15] and liver enzymes.[17] This analysis suggests that genetic variation at the *GCKR* locus may modulate NAFLD risk associated with obesity and/or elevated triglyceride levels. The same may be true for variants at the *LPL* locus, the gene that encodes lipoprotein lipase (LPL). LPL is a key enzyme that regulates the catabolism of triglycerides-rich lipoproteins such as chylomicrons and very-low-density lipoproteins in adipose tissue, skeletal muscle, and the heart. Gain-of-function mutations in LPL were associated with lower triglyceride levels and lower risk of CAD.[32] In the present study, we found a potentially causal inverse association between genetically predicted *LPL* expression in subcutaneous adipose tissue and NAFLD. These results are in line with the recent study of Maltais et al.,[33] who reported that 4 in 10 patients with familial chylomicronemia syndrome and almost 3 in 4 patients with multifactorial chylomicronemia syndrome

(2 disorders of impaired *LPL* function) met the criteria of NAFLD independently of their BMI. It should be noted that although the variant at the *LPL* locus linked with higher NAFLD was associated with higher liver enzymes levels in the UK Biobank, it was not associated with liver fat accumulation in the UK Biobank or with NAFLD in the Mass General Brigham Biobank. In addition, although these results did not reach the level of genome-wide significance, we found significant associations at the *MTARC1* and *MBOAT7* loci, thereby confirming the role of these genes in the etiology of NAFLD.

Previous studies have shown that NAFLD could be associated with or predict the risk of chronic diseases such as CVD or T2D. Our genetic correlation analyses revealed associations with these diseases as well as risk factors for these diseases such as obesity and insulin resistance. We also report interesting negative correlations between NAFLD and the ketone body acetoacetate (as previously suggested in an observational study),[34] as well as parental lifespan, suggesting that NAFLD may be a critical component of long-term disease risk potentially influencing human lifespan. Whether the resolution of NAFLD will influence these traits and outcomes remains to be determined. Interestingly, combined with the results of other studies that have linked variation at *LPL* as being associated with lower lipid levels and risk of CAD, our analysis suggests that targeting the LPL pathway may prevent NAFLD as well as other diseases such as hyperlipidemia and CAD without increasing the risk of other human diseases. Drugs targeting the LPL pathway under investigation for NAFLD include the angiopoietin-like protein-3 (ANGPTL3) inhibitors,[35] glucagon-like peptide-1 (GLP-1) receptor agonists,[36] and dual glucose-dependent insulinotropic peptide (GIP)/GLP-1 receptor agonists.[37] Drugs targeting obesity such as semaglutide were also recently associated with NASH resolution without worsening in liver fibrosis.[38]

Limitations of the study

Our study has limitations. For instance, although we have excluded secondary causes of NAFLD whenever possible, an EHR-based diagnosis of complex diseases such as NAFLD may be prone to misclassification of cases and controls. Our analysis revealed *FTO* and *LPL*, 2 potentially new NAFLD loci. However, although the top variants at these loci were associated with liver fat accumulation and/or liver enzymes in the UK Biobank, these variants did not replicate in a smaller NAFLD GWAS. It should also be re-emphasized that variation at these loci act on NAFLD through selected risk factors and therefore may lead to NAFLD via indirect mechanisms. Although our study reports 2 conventional GWAS analyses (adjusting or not adjusting for BMI), we could not perform a GWAS meta-analysis adjusting for triglyceride levels. Therefore, studies with larger sample sizes and accounting for triglyceride levels will be needed to document whether variation at the *LPL* locus are strongly associated with NAFLD and whether their effects are entirely mediated by triglyceride levels.

In conclusion, we conducted a large NAFLD GWAS based on EHRs from 4 cohorts to identify genetic variants of NAFLD susceptibility. We identified known NAFLD variants and show that variants associated with liver fat accumulation and liver enzymes may also be associated with the presence of NAFLD. Our analysis revealed a potentially causal effect of lower adipose-tissue expression of *LPL* and NAFLD that will need confirmation by other, larger studies.

STAR★METHODS

Detailed methods are provided in the online version of this paper and include the following:

- KEY RESOURCES TABLE
- RESOURCE AVAILABILITY
  - Lead Contact
  - Materials availability
  - Data and code availability
- EXPERIMENTAL MODEL AND SUBJECT DETAILS
  - Study participants
- METHOD DETAILS
- QUANTIFICATION AND STATISTICAL ANALYSIS
  - Genome-wide association study summary statistics NAFLD
  - Risk-factor informed Bayesian genome-wide association study
  - Transcriptome-wide association study of NAFLD
  - Replication of variants associated with NAFLD in the Mass General Brigham Biobank
  - Impact of NAFLD variants on liver fat accumulation in the UK Biobank
  - Impact of NAFLD variants on liver enzymes in the UK Biobank

SUPPLEMENTAL INFORMATION

Supplemental information can be found online at https://doi.org/10.1016/j.xcrm.2021.100437.

ACKNOWLEDGMENTS

We would like to thank all of the study participants as well as all of the investigators of the studies that were used throughout the course of this investigation (eMERGE, UK Biobank, and FinnGen). N.P. holds a doctoral research award from the Fonds de Recherche du Québec: Santé (FRQS). E.G. holds a master’s research award from FRQS. B.J.A. and S.T. hold junior scholar awards from the FRQS. P.M. holds a FRQS Research Chair on the Pathobiology of Calcific Aortic Valve Disease. Y.B. holds a Canada Research Chair in Genomics of Heart and Lung Diseases. M.-C.V. is Canada Research Chair in Genomics applied to Nutrition and Metabolic Health. Part of this study was supported by the European Union through the European Regional Development Fund. The work of the Estonian Genome Center, University of Tartu, has been supported by the European Regional Development Fund and grants GENTRANSMED (2014-2020.4.01.15-0012), MOBERA5 (Norface Network project no. 462.16.107), and 2014-2020.4.01.16-0125. This study was also funded by the European Union through the Horizon 2020 research and innovation programme under grant no. 810645 and through the European Regional Development Fund project no. MOBEC008 and Estonian Research Council grant no. PUT1660.

AUTHOR CONTRIBUTIONS

N.G., E.A., C.A.E., E. Gobeil, N.T., M.E.H., N.P., H.D.M., E. Gagnon, J.B., A.S-A., and C.C. analyzed and interpreted the data and created the tables and figures. N.G. and B.J.A. drafted the manuscript. All other authors provided critical revisions to the manuscript.

DECLARATION OF INTERESTS

A.T. receives research funding from Johnson & Johnson Medical Companies, Medtronic, Bodynov, and GI Windows for studies on bariatric surgery and has received consulting fees from Novo Nordisk and Bausch Health. A.V.K. has served as a scientific advisor to Sanofi, Amgen, Maze Therapeutics, Navitor Pharmaceuticals, Sarepta Therapeutics, Novartis, Verve Therapeutics, Silence Therapeutics, Veritas International, Color Health, Third Rock Ventures, and Columbia University (NIH); has received speaking fees from Illumina, MedGenome, Amgen, and the Novartis Institute for Biomedical Research; and has received sponsored research agreements from the Novartis Institute for Biomedical Research and IBM Research. B.J.A. is a consultant for Novartis and Silence Therapeutics and has received research contracts from Pfizer, Ionis Pharmaceuticals, and Silence Therapeutics.

INCLUSION AND DIVERSITY

We worked to ensure that the study questionnaires were prepared in an inclusive way. One or more of the authors self-identifies as an underrepresented ethnic minority in science.

Received: September 20, 2021
Revised: October 7, 2021
Accepted: October 12, 2021
Published: November 3, 2021

REFERENCES

1. Sumida, Y., and Yoneda, M. (2018). Current and future pharmacological therapies for NAFLD/NASH. J. Gastroenterol. *53*, 362–376.
2. Stefan, N., Häring, H.-U., and Cusi, K. (2019). Non-alcoholic fatty liver disease: causes, diagnosis, cardiometabolic consequences, and treatment strategies. Lancet Diabetes Endocrinol. *7*, 313–324.
3. Younossi, Z.M., Koenig, A.B., Abdelatif, D., Fazel, Y., Henry, L., and Wymer, M. (2016). Global epidemiology of nonalcoholic fatty liver disease-meta-analytic assessment of prevalence, incidence, and outcomes. Hepatology *64*, 73–84.
4. Eguchi, Y., Hyogo, H., Ono, M., Mizuta, T., Ono, N., Fujimoto, K., Chayama, K., and Saibara, T.; JSG-NAFLD (2012). Prevalence and associated metabolic factors of nonalcoholic fatty liver disease in the general population from 2009 to 2010 in Japan: a multicenter large retrospective study. J. Gastroenterol. *47*, 586–595.
5. Pais, R., Barritt, A.S., 4th, Calmus, Y., Scatton, O., Runge, T., Lebray, P., Poynard, T., Ratziu, V., and Conti, F. (2016). NAFLD and liver transplantation: current burden and expected challenges. J. Hepatol. *65*, 1245–1257.
6. Yoshitaka, H., Hamaguchi, M., Kojima, T., Fukuda, T., Ohbora, A., and Fukui, M. (2017). Nonoverweight nonalcoholic fatty liver disease and incident cardiovascular disease: a post hoc analysis of a cohort study. Medicine (Baltimore) *96*, e6712.
7. Brouwers, M.C.G.J., Simons, N., Stehouwer, C.D.A., and Isaacs, A. (2020). Non-alcoholic fatty liver disease and cardiovascular disease: assessing the evidence for causality. Diabetologia *63*, 253–260.
8. Kotronen, A., and Yki-Järvinen, H. (2008). Fatty liver: a novel component of the metabolic syndrome. Arterioscler. Thromb. Vasc. Biol. *28*, 27–38.
9. Targher, G., Day, C.P., and Bonora, E. (2010). Risk of cardiovascular disease in patients with nonalcoholic fatty liver disease. N. Engl. J. Med. *363*, 1341–1350.
10. Anstee, Q.M., Targher, G., and Day, C.P. (2013). Progression of NAFLD to diabetes mellitus, cardiovascular disease or cirrhosis. Nat. Rev. Gastroenterol. Hepatol. *10*, 330–344.
11. Lonardo, A., Ballestri, S., Marchesini, G., Angulo, P., and Loria, P. (2015). Nonalcoholic fatty liver disease: a precursor of the metabolic syndrome. Dig. Liver Dis. *47*, 181–190.
12. Neuschwander-Tetri, B.A., Clark, J.M., Bass, N.M., Van Natta, M.L., Unalp-Arida, A., Tonascia, J., Zein, C.O., Brunt, E.M., Kleiner, D.E., McCullough, A.J., et al.; NASH Clinical Research Network (2010). Clinical, laboratory and histological associations in adults with nonalcoholic fatty liver disease. Hepatology *52*, 913–924.
13. Kaps, L., Labenz, C., Galle, P.R., Weinmann-Menke, J., Kostev, K., and Schattenberg, J.M. (2020). Non-alcoholic fatty liver disease increases the risk of incident chronic kidney disease. United European Gastroenterol. J. *8*, 942–948.
14. Armstrong, M.J., Adams, L.A., Canbay, A., and Syn, W.K. (2014). Extrahepatic complications of nonalcoholic fatty liver disease. Hepatology *59*, 1174–1197.
15. Speliotes, E.K., Yerges-Armstrong, L.M., Wu, J., Hernaez, R., Kim, L.J., Palmer, C.D., Gudnason, V., Eiriksdottir, G., Garcia, M.E., Launer, L.J., et al.; NASH CRN; GIANT Consortium; MAGIC Investigators; GOLD Consortium (2011). Genome-wide association analysis identifies variants associated with nonalcoholic fatty liver disease that have distinct effects on metabolic traits. PLoS Genet. *7*, e1001324.
16. Parisinos, C.A., Wilman, H.R., Thomas, E.L., Kelly, M., Nicholls, R.C., McGonigle, J., Neubauer, S., Hingorani, A.D., Patel, R.S., Hemingway, H., et al. (2020). Genome-wide and Mendelian randomisation studies of liver MRI yield insights into the pathogenesis of steatohepatitis. J. Hepatol. *73*, 241–251.
17. Chambers, J.C., Zhang, W., Sehmi, J., Li, X., Wass, M.N., Van der Harst, P., Holm, H., Sanna, S., Kavousi, M., Baumeister, S.E., et al.; Alcohol Genome-wide Association (AlcGen) Consortium; Diabetes Genetics Replication and Meta-analyses (DIAGRAM+) Study; Genetic Investigation of Anthropometric Traits (GIANT) Consortium; Global Lipids Genetics Consortium; Genetics of Liver Disease (GOLD) Consortium; International Consortium for Blood Pressure (ICBP-GWAS); Meta-analyses of Glucose and Insulin-Related Traits Consortium (MAGIC) (2011). Genome-wide association study identifies loci influencing concentrations of liver enzymes in plasma. Nat. Genet. *43*, 1131–1138.
18. Emdin, C.A., Haas, M.E., Khera, A.V., Aragam, K., Chaffin, M., Klarin, D., Hindy, G., Jiang, L., Wei, W.Q., Feng, Q., et al.; Million Veteran Program (2020). A missense variant in Mitochondrial Amidoxime Reducing Component 1 gene and protection against liver disease. PLoS Genet. *16*, e1008629.
19. Anstee, Q.M., Darlay, R., Cockell, S., Meroni, M., Govaere, O., Tiniakos, D., Burt, A.D., Bedossa, P., Palmer, J., Liu, Y.L., et al.; EPoS Consortium Investigators (2020). Genome-wide association study of non-alcoholic fatty liver and steatohepatitis in a histologically characterised cohort☆. J. Hepatol. *73*, 505–515.
20. Mounier, N., and Kutalik, Z. (2020). bGWAS: an R package to perform Bayesian genome wide association studies. Bioinformatics *36*, 4374–4376.
21. Liu, Z., Zhang, Y., Graham, S., Wang, X., Cai, D., Huang, M., Pique-Regi, R., Dong, X.C., Chen, Y.E., Willer, C., and Liu, W. (2020). Causal relationships between NAFLD, T2D and obesity have implications for disease subphenotyping. J. Hepatol. *73*, 263–276.
22. Romeo, S., Kozlitina, J., Xing, C., Pertsemlidis, A., Cox, D., Pennacchio, L.A., Boerwinkle, E., Cohen, J.C., and Hobbs, H.H. (2008). Genetic variation in PNPLA3 confers susceptibility to nonalcoholic fatty liver disease. Nat. Genet. *40*, 1461–1465.
23. Kozlitina, J., Smagris, E., Stender, S., Nordestgaard, B.G., Zhou, H.H., Tybjærg-Hansen, A., Vogt, T.F., Hobbs, H.H., and Cohen, J.C. (2014). Exome-wide association study identifies a TM6SF2 variant that confers susceptibility to nonalcoholic fatty liver disease. Nat. Genet. *46*, 352–356.
24. Chen, V.L., Du, X., Chen, Y., Kuppa, A., Handelman, S.K., Vohnoutka, R.B., Peyser, P.A., Palmer, N.D., Bielak, L.F., Halligan, B., and Speliotes, E.K. (2021). Genome-wide association study of serum liver enzymes implicates diverse metabolic and liver pathology. Nat. Commun. *12*, 816.
25. Jamialahmadi, O., Mancina, R.M., Ciociola, E., Tavaglione, F., Luukkonen, P.K., Baselli, G., Malvestiti, F., Thuillier, D., Raverdy, V., Männistö, V., et al.

(2021). Exome-Wide Association Study on Alanine Aminotransferase Identifies Sequence Variants in the GPAM and APOE Associated With Fatty Liver Disease. Gastroenterology *160*, 1634–1646.e7.

26. Richardson, K., Nettleton, J.A., Rotllan, N., Tanaka, T., Smith, C.E., Lai, C.Q., Parnell, L.D., Lee, Y.C., Lahti, J., Lemaitre, R.N., et al. (2013). Gain-of-function lipoprotein lipase variant rs13702 modulates lipid traits through disruption of a microRNA-410 seed site. Am. J. Hum. Genet. *92*, 5–14.
27. GTEx Consortium (2015). Human genomics. The Genotype-Tissue Expression (GTEx) pilot analysis: multitissue gene regulation in humans. Science *348*, 648–660.
28. Liu, B., Gloudemans, M.J., Rao, A.S., Ingelsson, E., and Montgomery, S.B. (2019). Abundant associations with gene expression complicate GWAS follow-up. Nat. Genet. *51*, 768–769.
29. Haas, M.E., Pirruccello, J.P., Friedman, S.N., Emdin, C.A., Ajmera, V.H., Simon, T.G., Homburger, J.R., Guo, X., Budoff, M., Corey, K.E., et al. (2020). Machine learning enables new insights into clinical significance of and genetic contributions to liver fat accumulation. medRxiv. https://doi.org/10.1101/2020.09.03.20187195.
30. Vujkovic, M., Ramdas, S., Lorenz, K.M., Schneider, C.V., Park, J., Lee, K.M., Serper, M., Carr, R.M., Kaplan, D.E., Haas, M.E., et al. (2021). A genome-wide association study for nonalcoholic fatty liver disease identifies novel genetic loci and trait-relevant candidate genes in the Million Veteran Program. medRxiv. https://doi.org/10.1101/2020.12.26.20248491.
31. Scuteri, A., Sanna, S., Chen, W.M., Uda, M., Albai, G., Strait, J., Najjar, S., Nagaraja, R., Orrú, M., Usala, G., et al. (2007). Genome-wide association scan shows genetic variants in the FTO gene are associated with obesity-related traits. PLoS Genet. *3*, e115.
32. Stitziel, N.O., Stirrups, K.E., Masca, N.G., Erdmann, J., Ferrario, P.G., König, I.R., Weeke, P.E., Webb, T.R., Auer, P.L., Schick, U.M., et al.; Myocardial Infarction Genetics and CARDIoGRAM Exome Consortia Investigators (2016). Coding variation in ANGPTL4, LPL, and SVEP1 and the risk of coronary disease. N. Engl. J. Med. *374*, 1134–1144.
33. Maltais, M., Brisson, D., and Gaudet, D. (2021). Non-Alcoholic Fatty Liver in Patients with Chylomicronemia. J. Clin. Med. *10*, 669.
34. Männistö, V.T., Simonen, M., Hyysalo, J., Soininen, P., Kangas, A.J., Kaminska, D., Matte, A.K., Venesmaa, S., Käkelä, P., Kärjä, V., et al. (2015). Ketone body production is differentially altered in steatosis and non-alcoholic steatohepatitis in obese humans. Liver Int. *35*, 1853–1861.
35. Gaudet, D., Karwatowska-Prokopczuk, E., Baum, S.J., Hurh, E., Kingsbury, J., Bartlett, V.J., Figueroa, A.L., Piscitelli, P., Singleton, W., Witztum, J.L., et al.; Vupanorsen Study Investigators (2020). Vupanorsen, an N-acetyl galactosamine-conjugated antisense drug to ANGPTL3 mRNA, lowers triglycerides and atherogenic lipoproteins in patients with diabetes, hepatic steatosis, and hypertriglyceridaemia. Eur. Heart J. *41*, 3936–3945.
36. Vergès, B., Duvillard, L., Pais de Barros, J.P., Bouillet, B., Baillot-Rudoni, S., Rouland, A., Petit, J.M., Degrace, P., and Demizieux, L. (2021). Liraglutide Increases the Catabolism of Apolipoprotein B100-Containing Lipoproteins in Patients With Type 2 Diabetes and Reduces Proprotein Convertase Subtilisin/Kexin Type 9 Expression. Diabetes Care *44*, 1027–1037.
37. Wilson, J.M., Nikooienejad, A., Robins, D.A., Roell, W.C., Riesmeyer, J.S., Haupt, A., Duffin, K.L., Taskinen, M.R., and Ruotolo, G. (2020). The dual glucose-dependent insulinotropic peptide and glucagon-like peptide-1 receptor agonist, tirzepatide, improves lipoprotein biomarkers associated with insulin resistance and cardiovascular risk in patients with type 2 diabetes. Diabetes Obes. Metab. *22*, 2451–2459.
38. Newsome, P.N., Buchholtz, K., Cusi, K., Linder, M., Okanoue, T., Ratziu, V., Sanyal, A.J., Sejling, A.S., and Harrison, S.A.; NN9931-4296 Investigators (2021). A placebo-controlled trial of subcutaneous semaglutide in nonalcoholic steatohepatitis. N. Engl. J. Med. *384*, 1113–1124.
39. Zhou, W., Zhao, Z., Nielsen, J.B., Fritsche, L.G., LeFaive, J., Gagliano Taliun, S.A., Bi, W., Gabrielsen, M.E., Daly, M.J., Neale, B.M., et al. (2020). Scalable generalized linear mixed model for region-based association tests in large biobanks and cohorts. Nat. Genet. *52*, 634–639.
40. Willer, C.J., Li, Y., and Abecasis, G.R. (2010). METAL: fast and efficient meta-analysis of genomewide association scans. Bioinformatics *26*, 2190–2191.
41. Grotzinger, A.D., Rhemtulla, M., de Vlaming, R., Ritchie, S.J., Mallard, T.T., Hill, W.D., Ip, H.F., Marioni, R.E., McIntosh, A.M., Deary, I.J., et al. (2019). Genomic SEM provides insights into the multivariate genetic architecture of complex traits. Nat. Hum. Behav. *3*, 513–525.
42. Robinson, M.D., and Oshlack, A. (2010). A scaling normalization method for differential expression analysis of RNA-seq data. Genome Biol. *11*, R25.
43. Gamazon, E.R., Wheeler, H.E., Shah, K.P., Mozaffari, S.V., Aquino-Michaels, K., Carroll, R.J., Eyler, A.E., Denny, J.C., Nicolae, D.L., Cox, N.J., and Im, H.K.; GTEx Consortium (2015). A gene-based association method for mapping traits using reference transcriptome data. Nat. Genet. *47*, 1091–1098.
44. Barbeira, A.N., Dickinson, S.P., Bonazzola, R., Zheng, J., Wheeler, H.E., Torres, J.M., Torstenson, E.S., Shah, K.P., Garcia, T., Edwards, T.L., et al.; GTEx Consortium (2018). Exploring the phenotypic consequences of tissue specific gene expression variation inferred from GWAS summary statistics. Nat. Commun. *9*, 1825.
45. Bellenguez, C., Strange, A., Freeman, C., Donnelly, P., and Spencer, C.C.; Wellcome Trust Case Control Consortium (2012). A robust clustering algorithm for identifying problematic samples in genome-wide association studies. Bioinformatics *28*, 134–135.
46. Loh, P.-R., Tucker, G., Bulik-Sullivan, B.K., Vilhjálmsson, B.J., Finucane, H.K., Salem, R.M., Chasman, D.I., Ridker, P.M., Neale, B.M., Berger, B., et al. (2015). Efficient Bayesian mixed-model analysis increases association power in large cohorts. Nat. Genet. *47*, 284–290.
47. Kang, H.M., Sul, J.H., Service, S.K., Zaitlen, N.A., Kong, S.Y., Freimer, N.B., Sabatti, C., and Eskin, E. (2010). Variance component model to account for sample structure in genome-wide association studies. Nat. Genet. *42*, 348–354.
48. Namjou, B., Lingren, T., Huang, Y., Parameswaran, S., Cobb, B.L., Stanaway, I.B., Connolly, J.J., Mentch, F.D., Benoit, B., Niu, X., et al.; eMERGE Network (2019). GWAS and enrichment analyses of non-alcoholic fatty liver disease identify new trait-associated genes and pathways across eMERGE Network. BMC Med. *17*, 135.
49. GTEx Consortium (2020). The GTEx Consortium atlas of genetic regulatory effects across human tissues. Science *369*, 1318–1330.
50. Jongstra-Bilen, J., Haidari, M., Zhu, S.-N., Chen, M., Guha, D., and Cybulsky, M.I. (2006). Low-grade chronic inflammation in regions of the normal mouse arterial intima predisposed to atherosclerosis. J. Exp. Med. *203*, 2073–2083.
51. Karlson, E.W., Boutin, N.T., Hoffnagle, A.G., and Allen, N.L. (2016). Building the partners healthcare biobank at partners personalized medicine: informed consent, return of research results, recruitment lessons and operational considerations. J. Pers. Med. *6*, 2.
52. Zhou, W., Nielsen, J.B., Fritsche, L.G., Dey, R., Gabrielsen, M.E., Wolford, B.N., LeFaive, J., VandeHaar, P., Gagliano, S.A., Gifford, A., et al. (2018). Efficiently controlling for case-control imbalance and sample relatedness in large-scale genetic association studies. Nat. Genet. *50*, 1335–1341.
53. Wright, F.A., Sullivan, P.F., Brooks, A.I., Zou, F., Sun, W., Xia, K., Madar, V., Jansen, R., Chung, W., Zhou, Y.H., et al. (2014). Heritability and genomics of gene expression in peripheral blood. Nat. Genet. *46*, 430–437.
54. Littlejohns, T.J., Holliday, J., Gibson, L.M., Garratt, S., Oesingmann, N., Alfaro-Almagro, F., Bell, J.D., Boultwood, C., Collins, R., Conroy, M.C., et al. (2020). The UK Biobank imaging enhancement of 100,000 participants: rationale, data collection, management and future directions. Nat. Commun. *11*, 2624.
55. Bycroft, C., Freeman, C., Petkova, D., Band, G., Elliott, L.T., Sharp, K., Motyer, A., Vukcevic, D., Delaneau, O., O'Connell, J., et al. (2018). The UK Biobank resource with deep phenotyping and genomic data. Nature *562*, 203–209.

CellPress
OPEN ACCESS

STAR★METHODS

KEY RESOURCES TABLE

RESOURCE	SOURCE	IDENTIFIER
**Deposited data**		
Scripts	This paper	https://github.com/LaboArsenault
**Software and algorithms**		
SAIGE	Zhou et al.[39]	https://github.com/weizhouUMICH/SAIGE
METAL package	Willer et al.[40]	https://github.com/statgen/METAL
GenomicSEM R package	Grotzinger et al.[41]	https://github.com/GenomicSEM/GenomicSEM
STAR v2.6.1d	GENCODE v30	https://github.com/alexdobin/STAR
TMM (edgeR)	Robinson et al.[42]	https://www.biostars.org/p/317701/
S-PrediXcan	Gamazon et al.[43] and Barbeira et al.[44]	N/A
LocuscompareR (R package)	Liu et al.[28]	https://github.com/boxiangliu/locuscomparer
R package *aberrant*	Bellenguez et al.[45]	https://github.com/carbocation/aberrant
BOLT-LMM (version 2.3.4)	Loh et al.[46] and Kang et al.[47]	https://alkesgroup.broadinstitute.org/BOLT-LMM/BOLT-LMM_manual.html
bGWAS R package	Mounier et al.[20]	https://github.com/n-mounier/bGWAS
**Other**		
GWAS summary statistic of NAFLD (eMERGE)	Namjou et al.[48]	https://www.ebi.ac.uk/gwas/studies/GCST008468
GTEx consortium (version 8)	GTEx Consortium[49]	https://gtexportal.org/home/publicationsPage
GWAS summary statistics on liver enzymes (UK Biobank)	NA	http://www.nealelab.is/blog/2019/9/16/biomarkers-gwas-results
GWAS summary statistic for FinnGen	NA	https://www.finngen.fi/en/access_results
Research Ethics Committee of the University of Tartu	NA	Approval number 288/M-18
UK Biobank	NA	Data application number 25205

RESOURCE AVAILABILITY

Lead Contact

Further information and requests for resources and reagents should be directed to and will be fulfilled by the Lead Contact, Benoit Arsenault (benoit.arsenault@criucpq.ulaval.ca).

Materials availability

No materials were used to perform the genome-wide association meta-analysis and follow-up studies.

Data and code availability

GWAS summary statistics of the genome-wide meta-analysis of NAFLD have been deposited at the GWAS catalog and are publicly available as of the date of publication. The GWAS summary statistics for NAFLD of the eMERGE network are available at the GWAS catalog: https://www.ebi.ac.uk/gwas/studies/GCST008468.v The GWAS summary statistics for NAFLD of FinnGen are available here: https://www.finngen.fi/en/access_results. GWAS summary statistics on the liver enzymes measured in participants of the UK Biobank are available here: http://www.nealelab.is/blog/2019/9/16/biomarkers-gwas-results. DOIs are listed in the Key resources table.

All original code has been deposited at GitHub and is publicly available as of the date of publication: https://github.com/LaboArsenault. The bGWAS R package is available at: https://github.com/n-mounier/bGWAS. The *LocusCompareR* R package is available at https://github.com/boxiangliu/locuscomparer. The GenomicSEM R package is available at: https://github.com/GenomicSEM/GenomicSEM. DOIs are listed in the Key resources table.

Any additional information required to reanalyze the data reported in this paper is available from the lead contact upon request.

EXPERIMENTAL MODEL AND SUBJECT DETAILS

Study participants

To obtain a comprehensive set of NAFLD GWAS summary statistics, we performed a GWAS meta-analysis of four cohorts: The Electronic Medical Records and Genomics (eMERGE)[50] network, the UK Biobank, the Estonian Biobank and FinnGen. The NAFLD GWAS in the eMERGE network has previously been published. The study sample included 1106 NAFLD cases and 8571 controls participants of European ancestry. Of them, 396 NAFLD cases and 846 controls participants (47% males) were derived from a pediatric population and 710 NAFLD cases and 7725 controls participants (42% males) were derived from an adult population. NAFLD was defined by the use of EHR codes (ICD9: 571.5, ICD9: 571.8, ICD9: 571.9, ICD10: K75.81, ICD10: K76.0 and ICD10: K76.9. Exclusion criteria included, but were not limited to alcohol dependence, alcoholic liver disease, alpha-1 antitrypsin deficiency, Alagille syndrome, liver transplant, cystic fibrosis, hepatitis, abetalipoproteinemia, LCAT deficiency, lipodystrophy, disorders of copper metabolism Reye's syndrome, inborn errors of metabolism, HELLP syndrome, starvation and acute fatty liver (as suggested by the American Association for the Study of Liver Disease [AASLD]). We performed a new GWAS for NAFLD in the UK Biobank (data application number 25205). NAFLD diagnosis was established from hospital records (ICD10: K74.0 and K74.2 (hepatic fibrosis), K75.8 (NASH), K76.0 (NAFLD) and ICD10: K76.9 (other specified diseases of the liver). Exclusion criteria were the same as those used in the eMERGE study. In the UK Biobank analysis, we included 2558 NAFLD cases and 395,241 controls. We also performed a GWAS for NAFLD in the Estonian Biobank. This study and the use of data from 4119 cases and 190,120 controls was approved by the Research Ethics Committee of the University of Tartu (Approval number 288/M-18). We used the same case definition and inclusion/exclusion criteria as in the UK Biobank. In the FinnGen data freeze 4 (November 30, 2020), 651 patients had a NAFLD diagnosis (EHR code K76.0). They were compared to 176,248 controls. The Mass General Brigham Biobank is a hospital-based biorepository with genetic data linked to clinical records as previously described.[51] Patients were defined as having NAFLD or NASH according to diagnosis codes in the electronic health care record and were compared to controls without such diagnoses.

METHOD DETAILS

In the eMERGE study, logistic regression analysis was performed on over 7 million SNPs with MAF > 1% adjusted for age, gender, body mass index, genotyping site and the first three ancestry based principal components. In the UK Biobank genome-wide genotyping was available for over 28 million genetic markers directly genotyped or imputed by the Haplotype Reference Consortium (HRC) panel. In FinnGen, GWAS was performed using over 16 million genetic markers genotyped with the Illumina or Affymetrix arrays or imputed using the population specific SISu v3 reference panel. Variables included in the models were gender, age, the 10-main ancestry-based principal components and genotyping batch.

QUANTIFICATION AND STATISTICAL ANALYSIS

Genome-wide association study summary statistics NAFLD

We used the SAIGE (Scalable and Accurate Implementation of Generalized Mixed Models) method to perform the GWAS in the UK Biobank and in the Estonian Biobank[52]. This method is based on generalized mixed models and was developed to control for case-control imbalance, sample relatedness and population structure. In this analysis, gender, age and the 10 main ancestry-based principal components were used as covariates. Age, gender and the 10-main ancestry-based PCs were used as covariates. Finally, SAIGE was also used to obtain GWAS summary statistics of the FinnGen cohort. We performed a fixed-effect GWAS meta-analysis of the eMERGE, UK Biobank, FinnGen and Estonian Biobank cohorts using the METAL package.[40] When variants showed evidence of heterogeneity, we performed a random effect meta-analysis. A total of 6,797,908 SNPs with a minor allele frequency equal or above 0.01 were investigated. The genomic inflation factor and the LDSC intercept were computed using the GenomicSEM R package.[41]

Risk-factor informed Bayesian genome-wide association study

We used bGWAS to identify more SNPs associated with NAFLD.[20] The aim of bGWAS is to identify new variants associated with complex diseases using inference from risk factors of focal traits. We used GWAS summary statistics from two risk factors causally associated with NAFLD in a previous MR study[21] (BMI and triglyceride levels) as priors and worked with default parameters of the package as these two risk factors showed significant multivariable causal effects (Figure S2). The bWAS approach increases power over conventional GWAS by comparing the observed Z-statistics (the observed effect size for each SNP divided by its standard error) from the focal phenotype (i.e., NAFLD) to prior effects using Bayes Factors (Bayesian effects). The prior effects are calculated from publicly available GWAS summary statistics for related risk factors and are included in the bGWAS package. These were obtained from the Global Lipids Genetic Consortium and the Genetics of Anthropometric Traits (GIANT). Briefly, bGWAS derives informative prior effects from these risk factors and their causal effect on NAFLD using multivariable MR. Prior estimates (mu) are calculated for each SNP by multiplying the SNP-risk factor effect by the risk factor-NAFLD causal effect estimates. By combining observed effects from the NAFLD GWAS meta-analysis and prior effects, Bayes factors, posterior effects and direct effects and their corresponding p

values are generated. The direct effect of each SNP is the part of the observed effect that is not mediated through the selected risk factors.

Transcriptome-wide association study of NAFLD

Tissues from the GTEx consortium (version 8) with less than 70 samples were not used to provide sufficient statistical power for eQTL discovery, resulting in a set of 48 tissues. Only non-gender-specific tissues (N = 43) were analyzed. Alignment to the human reference genome hg28/GRCh38 was performed using STAR v2.6.1d, based on the GENCODE v30 annotation. RNA-seq expression outliers were excluded using a multidimensional extension of the statistic described by Wright et al.[53] Samples with less than 10 million mapped reads were removed. For samples with replicates, replicate with the greatest number of reads were selected. Expression values were normalized between samples using TMM as implemented in edgeR.[42] For each gene, expression values were normalized across samples using an inverse normal transformation. eQTL prediction models were performed using elastic net, a regularized regression method, as implemented in S-PrediXcan.[43,44] We used SNPs with a minor allele frequency greater than 1% from European ancestry participants. *Locuscompare* function from the *LocuscompareR* R package[28] was used to depict the colocalization event at the *LPL* locus. *Locuscompare* enables visualization of the strengths of eQTLs and outcomes associations by plotting p values for each within a given genomic location, thereby contributing to distinguish candidates from false-positive genes.

Replication of variants associated with NAFLD in the Mass General Brigham Biobank

In this cohort, genotyping was performed using the Illumina MEGA array. Association of each of the seven variants associated with NAFLD was assessed using logistic regression of disease status with age, gender and five principal components of ancestry as covariates.

Impact of NAFLD variants on liver fat accumulation in the UK Biobank

As part of the study protocol of the UK Biobank, a subset of individuals who underwent detailed imaging between years 2014 and 2019 including abdominal MRI.[54] Liver fat in this cohort was quantified via machine learning of abdominal MRI images as previously described.[29] We excluded samples that had no imputed genetic data, a genotyping call rate < 0.98, a mismatch between submitted and inferred gender, sex chromosome aneuploidy, exclusion from kinship inference, excessive third-degree relatives, or that were outliers in heterozygosity or genotype missingness rates, all of which were previously defined centrally by the UK Biobank[55] Due to the small percentage of samples of non-European ancestries, to avoid artifacts from population stratification we restricted our GWAS to samples of European ancestries, determined via self-reported ancestry of British, Irish, or other white and outlier detection using the R package *aberrant*, resulting in a total of 32,976 individuals. We did not remove related individuals from this analysis as we used a linear mixed model able to account for cryptic relatedness in common variant association studies.[46] For analysis of liver fat as a continuous trait, we applied a rank-based inverse normal transformation. We took the residuals of liver fat in a linear model that included gender, year of birth, age at time of MRI, age at time of MRI squared, genotyping array, MRI device serial number, and the first ten principal components of ancestry. We then performed the inverse normal transform on the residuals from this model, yielding a standardized output with mean 0 and standard deviation of 1. We measured the association of genetic variants with rank inverse normal transformed liver fat via a linear mixed model using BOLT-LMM (version 2.3.4) to account for ancestry, cryptic population structure, and sample relatedness. The default European linkage disequilibrium panel provided with BOLT was used.

Impact of NAFLD variants on liver enzymes in the UK Biobank

Age, gender and ancestry-based principal components-adjusted GWAS summary statistics on ALT, AST, GGT and ALP concentrations in 361,194 participants of the UK Biobank of European ancestry were obtained from the Neale lab. Details on the protocols used to measure these biomarkers is available on the UK Biobank website: https://biobank.ndph.ox.ac.uk/showcase/showcase/docs/serum_biochemistry.pdf.

Cell Reports Medicine, Volume *2*

Supplemental information

Electronic health record-based genome-wide meta-analysis provides insights on the genetic architecture of non-alcoholic fatty liver disease

Nooshin Ghodsian, Erik Abner, Connor A. Emdin, Émilie Gobeil, Nele Taba, Mary E. Haas, Nicolas Perrot, Hasanga D. Manikpurage, Éloi Gagnon, Jérôme Bourgault, Alexis St-Amand, Christian Couture, Patricia L. Mitchell, Yohan Bossé, Patrick Mathieu, Marie-Claude Vohl, André Tchernof, Sébastien Thériault, Amit V. Khera, Tõnu Esko, and Benoit J. Arsenault

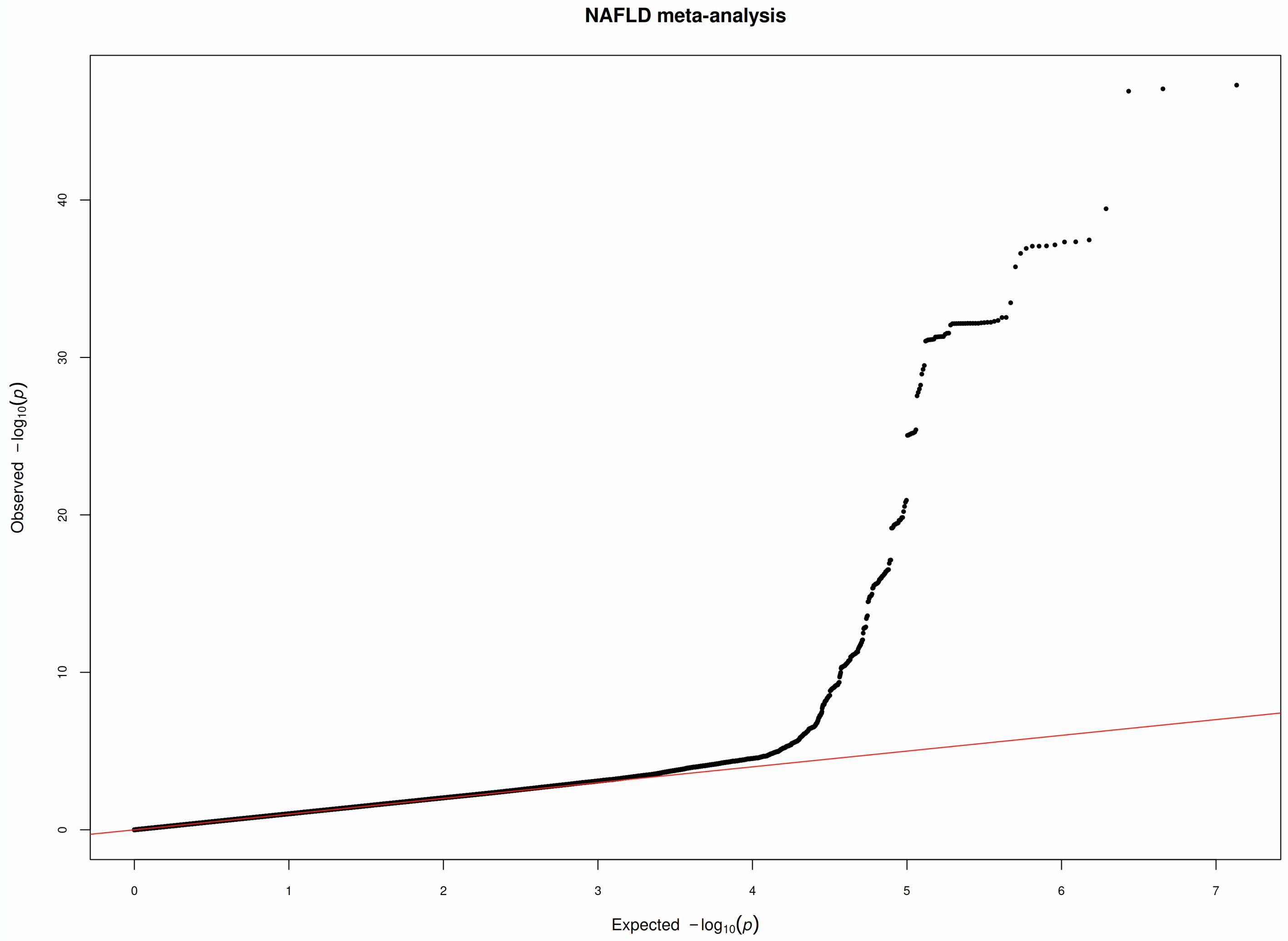

Supplementary Figure 1: Quantile-quantile plot of the NAFLD GWAS meta-analysis.

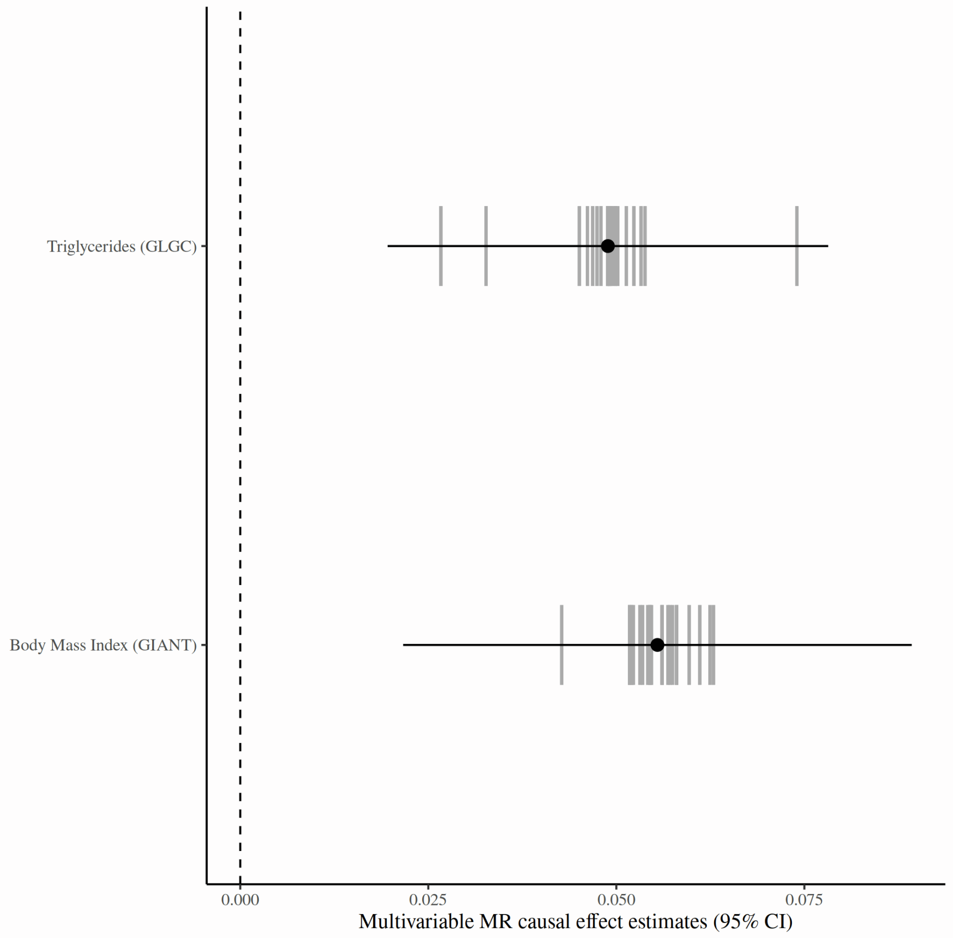

Supplementary Figure 2: Coefficient plot for the risk factors used to create the prior. For each risk factor, the multivariable causal estimate and the 95% interval from multivariable Mendelian randomization model using all chromosome (black dots and bars) as well as the 22 per-chromosome estimates (grey bars) are presented.

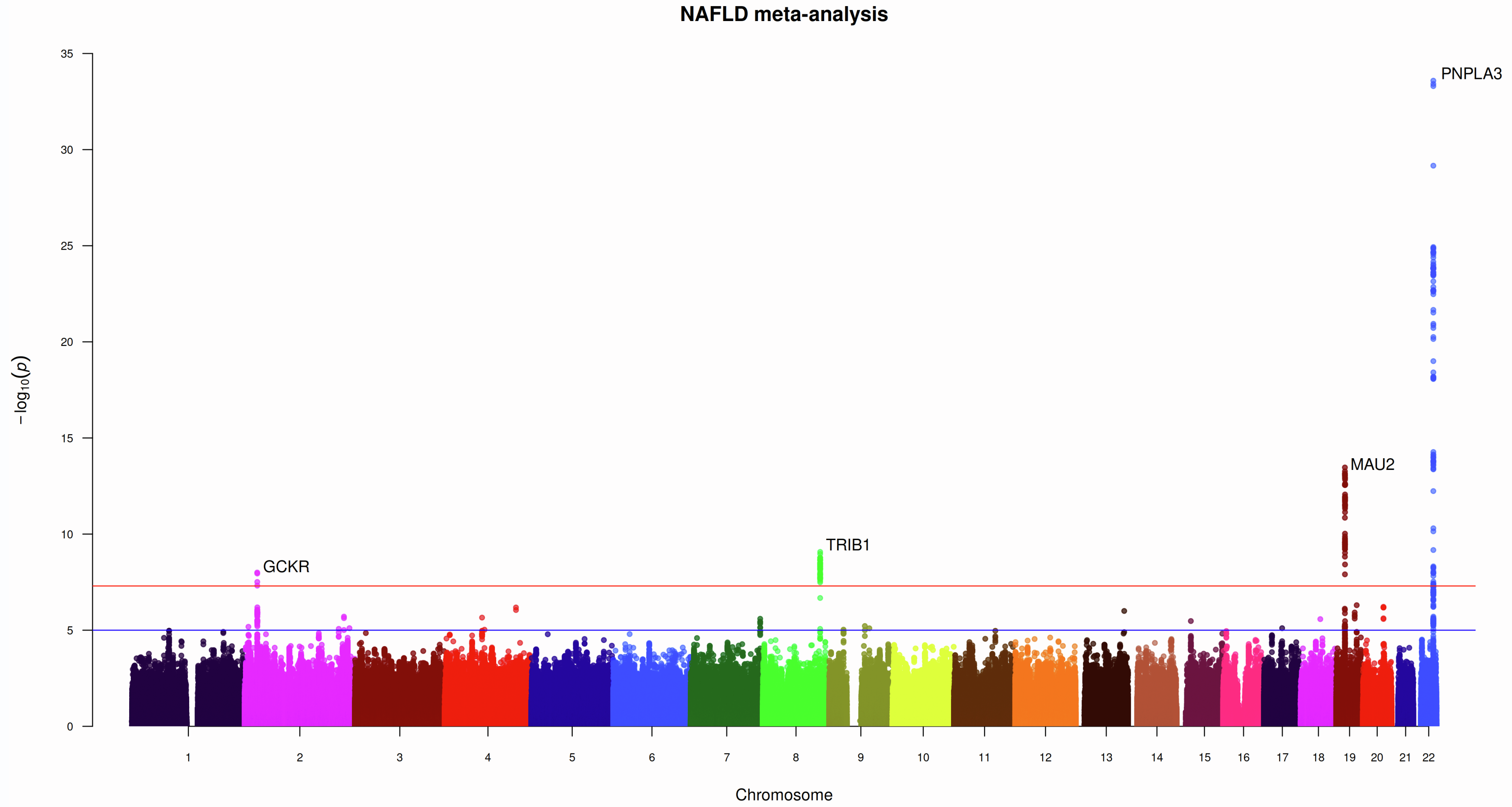

Supplementary Figure 3: Results of body-mass index-adjusted meta-analysis of genome-wide association studies.
